# Nucleation of the Theophylline:Salicylic Acid 1:1 Cocrystal

**DOI:** 10.1021/acs.cgd.0c01594

**Published:** 2021-04-01

**Authors:** Hannah McTague, Åke C. Rasmuson

**Affiliations:** † Synthesis and Solid State Pharmaceutical Centre (SSPC), Bernal Institute, Department of Chemical and Environmental Science, University of Limerick, Limerick V94 T9PX, Ireland; ‡ Department of Chemical Engineering and Technology, KTH Royal Institute of Technology, SE-100 44 Stockholm, Sweden

## Abstract

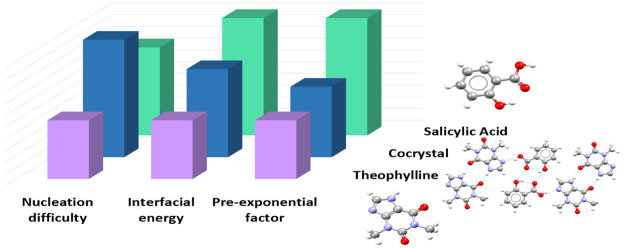

The nucleation behavior of the theophylline–salicylic acid 1:1 (THP:SA) cocrystal in chloroform has been investigated and compared with the corresponding behavior of the pure compounds. Induction times have been determined at different supersaturations at 10 °C under each condition in approximately 40–80 repetition experiments in 20 mL vials. Nucleation times, extracted from the median induction times by accounting for a nucleus growth time, have been used to determine the interfacial energy and the pre-exponential factor within the classical nucleation theory. Results show that the cocrystal at equal driving force has a longer nucleation time, or to reach equal nucleation time, the cocrystal requires a higher driving force. Pure theophylline is easier to nucleate than pure salicylic acid, despite the latter having a smaller molecular size, higher solubility, and is expected to form dimers already in the solution. The cocrystal is found to have an interfacial energy in between the respective values for the pure compounds. However, the higher molecular volume of the cocrystal, taken as the volume of the 1:1 theophylline–salicylic acid assembly, leads to the highest nucleation work, which, together with a low pre-exponential factor, explains why the cocrystal is the most difficult to nucleate. The experimentally extracted pre-exponential factor of the cocrystal is very similar to that of THP, and similar trends are observed from theoretical expressions of volume-diffusion- and surface-integration-controlled nucleation, respectively.

## Abstract 2

Induction time experiments performed on the theophylline:salicylic acid 1:1 cocrystal and the pure components reveal that the cocrystal exhibits the highest “nucleation difficulty” followed by salicylic acid and theophylline, respectively. The observed nucleation difficulty is investigated through the application of classical nucleation theory and analysis of nucleation parameters including interfacial energy and pre-exponential factor.

## Introduction

In recent times, the pharmaceutical industry has reappraised a long discovered but relatively little applied alternative crystal form, a cocrystal.^1−5^ A cocrystal is a crystalline solid consisting of two or more different molecular compounds bonded intermolecularly, typically through H bonds.^6^ If one or more of the constituent molecules have a therapeutic effect, it can be classified as a pharmaceutical cocrystal.^7^ Approximately 40% of all drug molecules on the market display undesirable physicochemical properties (PCP) such as low solubility and dissolution rates and poor thermal stability and bioavailability.^8^ A cocrystal has the potential to offer an alternative form of an active pharmaceutical ingredient (API), one that may display improved physicochemical properties and enhanced processability while the chemical form remains unchanged.^1,2,5,9,10^ Another attractive feature of cocrystals to the pharmaceutical industry is that they are amenable to design by crystal engineering. Traditionally, APIs have been confined to crystalline forms such as salts, polymorphs, solvates, and hydrates. In cases where APIs have limited solubility and lack ionizable functional groups for salt formation, a solution may be offered in the form of cocrystallization.^6,11^

Although cocrystals have been long discovered, there is limited work on their physical properties and processing, especially the conditions suitable for manufacturing at an industrial scale. Thermodynamics of cocrystals have been explored in the literature to some extent;^12−17^ however, few studies have been performed examining the crystallization kinetics of cocrystals, and, to our knowledge, no investigation into the actual kinetics of cocrystal nucleation has been reported.

There are a significant number of studies on the nucleation of pure compounds. However, the understanding of nucleation mechanisms is still very unsatisfactory. In most studies, the quantitative experimental data are evaluated by the classical nucleation theory (CNT), even though it has been found repeatedly that the parameters calculated appear to be unrealistic, e.g., the nucleation work is very low and the critical nucleus size is very small. In most studies, the nucleation of a single compound, perhaps in a few different solvents, is investigated. In a few studies, though, the nucleation of different solid phases has been compared,^18,19^ and it has been found that even if nucleation is regarded to be very case sensitive and stochastic, the data suggest that a certain rationalization can be made. Concerning the nucleation of cocrystals, very few studies have been published. In the nucleation of the *p*-toluenesulfonamide/triphenylphosphine oxide cocrystal,^17^ it was found that kinetic factors influence the form of the nucleating phase. To our knowledge, there is no previous study in which the nucleation of a cocrystal has been investigated quantitatively and systematically and the result has been compared with the nucleation behavior of the corresponding pure compounds.

**Figure 1:**
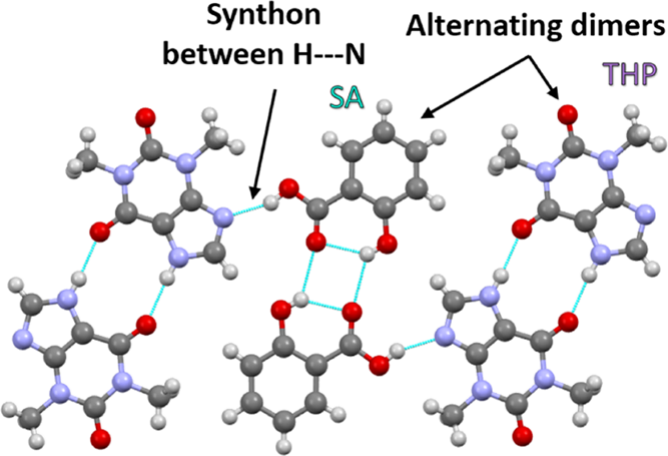
THP:SA cocrystal.

In the present work, we present a quantitative investigation of the nucleation of the 1:1 cocrystal of theophylline–salicylic acid (THP:SA) in chloroform shown in Figure 1. The purpose is to explore if the nucleation of a cocrystal shows principal differences to the nucleation of pure compounds and if the nucleation of the cocrystal can be rationally related to the nucleation of the corresponding pure compounds. The phase diagrams of the 1:1 THP:SA cocrystal in different solvents have been reported.^12^ In chloroform, the cocrystal dissolves congruently, and this is an important reason for choosing this solvent, even though the volatility will require special care in the experimentation. There is one reported crystal structure of the cocrystal, and the unit cell contains four THP molecules and four SA molecules arranged in homopairs of centrosymmetric dimers. The alternating dimers are bonded by hydrogen bonds through the hydrogen of the carboxylic acid group on SA and the nitrogen of the THP 5-ring.^12^ Theophylline is a methylxanthine derivative with diuretic properties and is a bronchodilator commonly indicated for the treatment of asthma.^20^ Salicylic acid has bacteriostatic, keratolytic, and fungicidal properties. Previously, salts of SA were used as analgesic drugs; however, nowadays, SA is advised for the treatment of acne.^21^

THP is known to appear in five different polymorphic forms thus far, where the crystal form IV based on THP dimers has been found to be the thermodynamically stable form.^22^ THP form II is the polymorph most commonly observed to crystallize from nonaqueous solutions and does not contain this dimer motif.^23,24^ Only one crystal structure has been reported for salicylic acid.

The crystal nucleation of salicylic acid in a range of solvents by induction time experiments and metastable zone width measurements has been previously investigated.^25^ It has been observed that as calculated interfacial energies increase, the induction times at equal driving force also increase.^26^ A further investigation into the effect of different solvents on SA nucleation by means of spectroscopy in conjunction with density functional theory (DFT) calculations revealed a correlation between the strength of solvent–solute interactions and difficulty of nucleation.^25^

The crystal nucleation of hydrated and anhydrous theophylline has also received some attention. Details of the transformation kinetics described a method by which the anhydrous form nucleates first, acting as a heterogeneous substrate for the monohydrate form to grow on, thus hindering subsequent dissolution of the anhydrous metastable form.^27^ More recently, a study revealed that the self-mediated transformation of polymorphic form theophylline from II to IV is nucleation-growth-controlled through the self-association effect. The study unveiled a relationship between solvent-mediated phase transformation induction times and phase transition times. It was observed how solution aggregates of theophylline hinder phase transformation; however, the induction time is not dependent on aggregation kinetics but rather on the hydrogen bond amenability of the solvent.^28^

## Experimental Section

Nucleation experiments have been carried out in chloroform for the THP:SA cocrystal and for pure compounds, respectively. The solubility of the cocrystal, THP II, and SA has been determined at the nucleation temperature.

### Materials

Theophylline (THP, CAS Registry Number 58-55-9) was used as received from Sigma-Aldrich in the form of an anhydrous powder with purity ≥99%. Salicylic acid (SA, CAS Registry Number 69-72-7) with purity ≥99% was used as received from Sigma-Aldrich. Chloroform (CLO, CAS Registry Number 67-66-3) had high-performance liquid chromatography (HPLC) purity ≥99.9%. The solvent was maintained in well-sealed bottles at all times and used as received from Merck.

For manufacturing of the cocrystal, THP II and SA (equimolar) are added to chloroform and stirred at 200 rpm, forming a slurry. The system is sealed and stirred continuously for 72 h. Following slow slurry conversion, the solid phase is isolated and dried by evaporation. Three samples are taken from the bulk mass and analyzed by powder X-ray diffraction (PXRD). Following physical characterization and determination, the desired cocrystal is then stored for use in induction time experiments.

### Procedures

The supersaturation is calculated as a mole fraction ratio
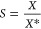
1where *X* is the mole fraction of the solute in the solution and X* is the mole fraction solubility at the nucleation temperature (moles of solute/moles total). For the THP:SA cocrystal system, the supersaturation was calculated according to
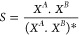
2where *X*^*A*^ is the mole fraction concentration of THP II, *X*^*B*^ is the mole fraction concentration of SA in the solution, and the denominator represents the solubility product at the nucleation temperature. Pure THP:SA cocrystal was weighed and added to chloroform to create solutions of the desired concentration for cocrystal induction time experiments.

For the nucleation experiments, the solution is prepared by weighing with the desired amount of solid phase and solvent and the solid is allowed to dissolve completely at 50 °C. The solution is allowed to equilibrate for 24 h while stirring at 400 rpm. The solution is then aliquoted into 20 preheated 30 mL glass vials and sealed immediately with poly(tetrafluoroethylene) (PTFE)-coated caps to minimize evaporation. To avoid crystallization, the entire apparatus is heated in advance. A filtered 20 mL sample of the stock is added to each 30 mL glass vial containing a stir bar, the vials containing the liquid are placed in a 50 °C water bath, and the liquid is stirred at 400 rpm for 24 h. Supersaturation is created by transferring the vials to a water bath at a nucleation temperature of 10 ± <0.1 °C.

The vials were recorded on an HD video camera from the time they were submerged at 10 °C up until all vials showed nucleation. After nucleation, the vials were brought to the dissolution temperature water bath and the solutions were allowed to fully dissolve for 24 h, before again placing the vials in the nucleation water bath for a second nucleation experiment. Each set of vials was subjected to the nucleation cycle twice. The vials were weighed before and after experiments to ensure that evaporation losses were negligible. If the weights varied by more than 0.05%, the induction times were not included.

This was then repeated for a range of different concentrations by which different supersaturations were covered. Induction times were taken as the amount of time elapsed following the placement of the vials into the nucleation temperature water baths and the first detection of crystals in solution. The HD video recordings were rigorously analyzed by the naked eye, and the time taken for the first visible nucleation was identified and recorded manually. A nucleation event is determined by the sudden presence of a cloud point where the solution is no longer transparent. An identical vial containing only chloroform was included in some batches and used to check the temperature with a digital thermometer at various stages of the nucleation cycle. The temperature checks showed that vials would reach the nucleation temperature (10 ± <0.1 °C) in less than 2 min and no nucleation event was recorded prior to this time.

It is obvious that at the same supersaturation, there exists a large variation in induction times. Individual vial induction time showed variability during batch duplication; however, consistency was observed in the median induction time (τ_50_) for batches of the same degree of supersaturation. For certain concentrations in this work, 80 repetition experiments were performed. The τ_50_ was compared to that observed after 40 experiments, exhibiting negligible change following the extra experiments, typically <100 s difference. In fact, a very small change in τ_50_ was observed on the basis of 20 versus 40 experiments. For example, at *S* = 1.57, for the THP:SA system, τ_50_ was 3481 s based on the first 20 experiments and 3131 s based on the second set of experiments; in all 40 experiments, the τ_50_ was identified as 3280 s. This change in τ_50_ demonstrated almost no effect on the subsequently calculated nucleation parameters, interfacial energy, and pre-exponential kinetic factors.

There is no evidence that the cocrystal formation is preceded by nucleation of any pure component solid phase on which the nucleation of the cocrystal then occurs. No peaks of pure components can be found in any of the cocrystal nucleation experiments. The only solid identified by PXRD upon filtration promptly succeeding nucleation is a pure cocrystal.

The solubility was determined for SA, THP II, and THP:SA in chloroform at the nucleation temperature of 10 °C by the gravimetric method, the details of which are presented in the Supporting Information.

Techniques employed for characterization of the solid phase include PXRD, differential scanning calorimetry (DSC), thermogravimetric analysis (TGA), single-crystal X-ray diffraction (SC-XRD) analysis, and scanning electron microscopy (SEM), which are described in detail in the Supporting Information.

## Results and Analysis

### Solid-Phase Characterization and Solid–Liquid Solubility

The THP:SA cocrystal was successfully synthesized and was identified through PXRD as the previously reported form entered into the Cambridge structural database (CSD) under the reference code KIGLES01. The same solid form was also identified as the form being generated in the THP:SA induction time experiments, proving that the cocrystal is the nucleating solid. Following the induction time experiments, theophylline solid was identified as the polymorphic form II (CSD ref code BAPLOT01) and the salicylic acid solid material as the only known form (CSD ref code SALIAC). The melting point of the cocrystal was determined to be 186.2 °C, which was slightly lower than the previously published value, and the melting enthalpy was found to be 46.142 kJ mol^–1^ (per mole cocrystal assembly, i.e., one molecule of each of the compounds). Per gram solid material, the THP:SA cocrystal has a lower enthalpy of melting than the pure compounds. The cocrystal forms needle-shaped crystals as does salicylic acid, while the THP II crystals are platelike. Further details, powder diffractograms, scanning calorimetry profiles, and photographs are given in the Supporting Information.

The solubilities of THP:SA, SA, and THP II determined in chloroform at nucleation temperature (*T*_nuc_) are given in Table 1.

**Table 1:** Solubility of THP:SA, THP II, and SA in Chloroform at 10.0 °C

	g solute/g solvent	std. dev.	no. experiments	solubility (mole fraction)	mol m^–3^
THP:SA	0.00249	7.74 × 10^–5^	8	9.35 × 10^–4^	11.70
SA	0.00628	3.11 × 10^–4^	6	5.34 × 10^–3^	67.93
THP II	0.00205	6.25 × 10^–5^	13	1.39 × 10^–3^	17.36

The g solute/g solvent is the solubility directly obtained from the gravimetric solubility determination method. The cocrystal mole fraction solubility is calculated from the g/g measurement as the moles of the cocrystal 1:1 assembly (defined as 1 mole of THP and 1 mole of SA) per the total moles of the cocrystal 1:1 assembly and solvent. The solubility product of the cocrystal at 10.0 °C is 8.8867 × 10^–7^ (mol/mol)^2^. THP II exhibits a lower mole fraction solubility in chloroform than SA, and the THP:SA cocrystal has the lowest mole fraction solubility of the three solid phases. Similar cases have been reported previously in studies where the cocrystal displayed solubility outside of the range of the two coformers.^15,29,30^

### Nucleation Kinetics

During induction time experiments, the nucleating solutions were observed to transform from transparent to slightly cloudy and then completely opaque over time depending on the driving force. For the cocrystal, the time lapse between the first sign of cloudiness and maximum opacity was in the range of 1 h for the higher *S* values while taking up to 3 h for lower degrees of supersaturation. This is a very long time compared to the behavior of the pure systems. During nucleation of SA, total opacity ensues 10–30 s after the first detection of crystals by the naked eye. In THP II, full opacity was reached within a period of 3–6 min. Higher supersaturation leads to more rapid transformation from a clear to a totally opaque solution once nucleation occurs. The time of reaching full opacity from the very first sign of nucleation decreases among the three solid forms in the same order as the solubility increases. Nucleated vials in the THP II experiments were filtered immediately after nucleation to verify by PXRD that the polymorphic form was form II (Supporting Information).

**Figure 2:**
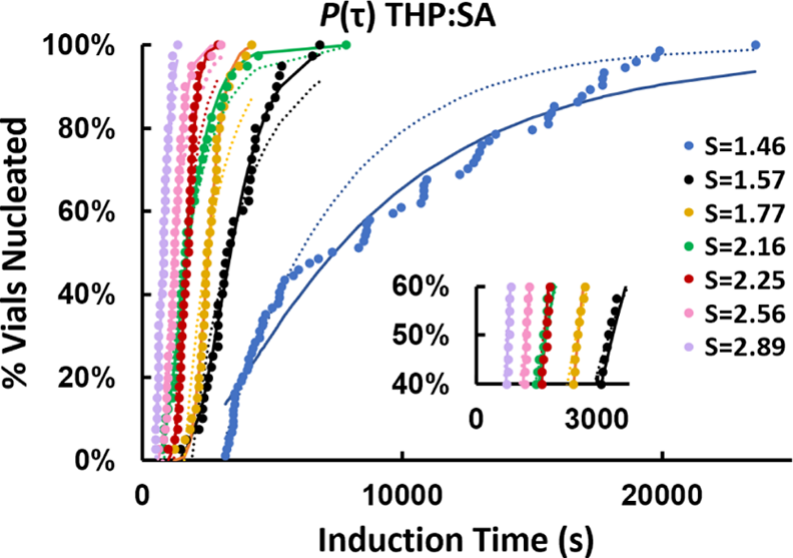
Induction time probability distributions, *P*(τ), for THP:SA at different supersaturation ratios (*S*) ranging from 1.46 to 2.89. The solid and dashed lines show log-normal and Poisson distribution functions, respectively, fitted to the experimental data. The magnified image of *P*(τ) is shown in the lower right part.

**Figure 3:**
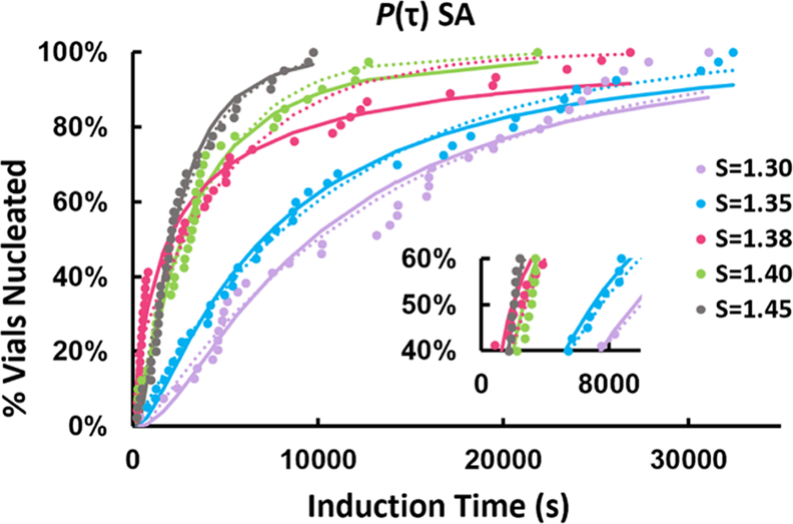
*P*(τ) for SA experimentally determined in chloroform at a nucleation temperature of 10 °C at various supersaturation ratios (*S*). The solid and dotted lines show log-normal and Poisson distributions, respectively, fitted to the experimental data. The magnified image is shown in the lower right part to visualize the smoothing effect of the Poisson distribution (dotted line) on τ_50_ at *S* = 1.38 (pink) and *S* = 1.40 (green).

**Figure 4:**
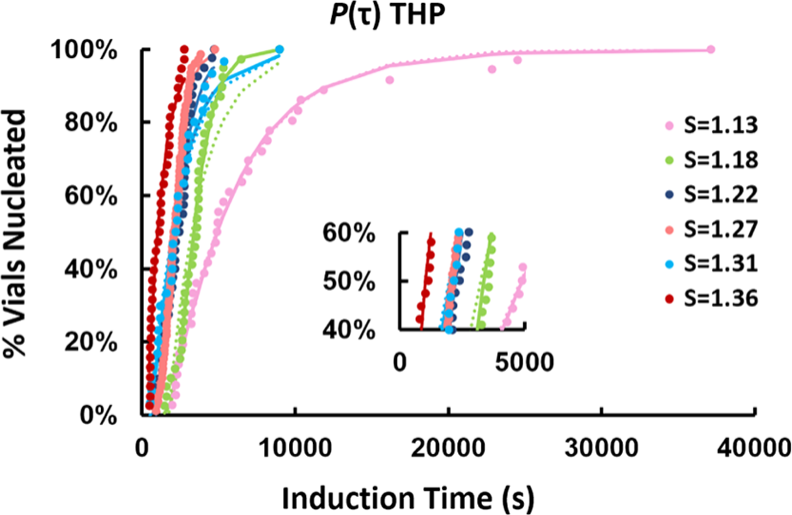
*P*(τ) for THP II experimentally determined in chloroform at a nucleation temperature of 10 °C at various supersaturation ratios (*S*). The solid and dotted lines show log-normal and Poisson distributions, respectively, fitted to the experimental data. The magnified image is shown in the lower right part to visualize the smoothing effect of the Poisson distribution (dotted line) on τ_50_ at *S* = 1.31 (pale blue).

The induction time (τ) probability distributions determined for the cocrystal, salicylic acid, and theophylline are shown in, respectively, Figures 2, 3, and 4, together with fittings by two different distribution functions: a Poisson distribution (eq 3)^31^ and a log-normal cumulative distribution function (eq 4)
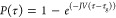
3
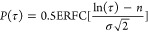
4where *V* is the sample volume, τ is the induction time, τ_g_ is the nucleus growth time, *n* is the location parameter, σ is the scale parameter, and τ is the individual induction time measurement. To be visible, nuclei have to grow to a detectable size. The time lapse between the time of actual nucleation and the time of detection is referred to as growth time, τ_g_, which is accounted for in eq 3. Both distributions fit reasonably well to all data with coefficients of determination (*R*^2^) values >0.9 in most cases. There was no noticeable difference between the functions in terms of compatibility with the experimental data. In some cases, such as *S* = 1.38 for SA (Figure 3), neither distribution fits the data well toward the upper end of the curve. In other cases, such as *S* = 1.46 for THP:SA, the Poisson function fits (Figure 2) the curve nicely in the beginning and at the end, however, the log-normal distribution gave a better representation of the middle section of data.

Obviously, for all three solid phases, there is a substantial spread in the induction times under each condition, presented in Table 2 as a coefficient of variation (CV) around a mean value. There is a tendency for CV to be higher for SA, but the values are quite high for all systems.

**Table 2:** Median Induction Times, τ_50_ (s), Obtained from Experimentally Determined Probability Distributions over a Range of Supersaturation Ratios, *S*, with Corresponding Driving Forces (RTln*S*) for THP:SA, SA, and THP II Systems^a^

THP:SA					SA					THP				
*S*	RT ln *S* (J mol^–1^)	τ_50_ (s)	CV	τ_g_ (s)	*S*	RT ln *S* (J mol^–1^)	τ_50_ (s)	CV	τ_g_ (s)	*S*	RT ln *S* (J mol^–1^)	τ_50_ (s)	CV	τ_g_ (s)
1.46	939	7326	0.60	3205	1.30	581	10186	0.68	338	1.13	349	4885	0.98	1980
1.57	1106	3280	0.33	1497	1.35	671	7246	0.86	205	1.18	449	3566	0.39	1471
1.77	1384	2505	0.22	1216	1.38	724	2774^b^	1.28	195	1.22	525	2417	0.40	710
2.16	1856	1735^c^	0.61	735	1.40	758	2751^b^	1.03	188	1.27	618	2100	0.33	870
2.25	1951	1747	0.20	992	1.45	842	2020	0.83	210	1.31	707	1960^b^	0.69	530
2.56	2259	1245	0.34	612						1.36	792	1160	0.57	445
2.89	2507	816	0.26	530										

a Growth times, τ_g_ (s), as the first induction time point; coefficient of variation (CV) (standard deviation/mean) calculated to describe the spread of nucleation induction times under each condition of supersaturation for THP:SA, SA, and THP II.

b Except for τ_50_ as per the Poisson fit to data.

c Except for τ_50_ as per the log-normal fit to data.

From the experimentally determined series of induction times, a median induction time (τ_50_) is obtained for each supersaturation driving force, shown in Table 2. The median induction time τ_50_ was extracted directly from the experimental measurements, except for in four cases where this reading became uncertain and data smoothing was required. For these four cases, the fitting of the function used was excellent in the τ_50_ range.

Application of the Poisson distribution,^31^eq 3, to the induction time data enabled the determination of growth times, τ_g_, for each system, except in the case of *S* = 1.38 for SA, where a negative τ_g_ value was obtained (Supporting Information). In general, as supersaturation increases, the τ_g_ values tend to decrease for all systems (Table 2). The THP:SA cocrystal system displayed the longest τ_g_ times for the range of supersaturation in this study, and for this system, the growth time is not negligible compared to the median distribution time. SA presented the shortest growth times and THP had intermediate τ_g_ values, which correlate with the evolution of opacity in the three systems as described. Besides the negative τ_g_ value, sometimes, the Poisson equation does not fit the experimental data well in the lower end of the distribution. In the present study, the τ_g_ value has instead been set as the time of the first induction time point of each distribution, which often is quite similar to the value obtained from eq 3 but alleviates the problems mentioned above. Henceforth, the growth time (τ_g_) refers to the induction time of the first point of each distribution unless otherwise specified. A comparison of the nucleation parameters calculated using the τ_g_ value from the Poisson fit can also be found in the Supporting Information.

**Figure 5:**
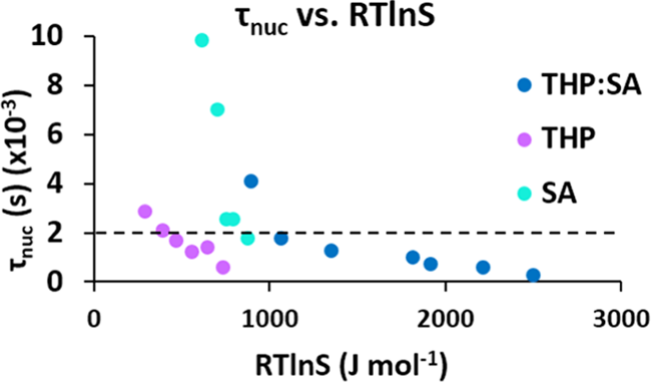
Graph displaying three systems THP:SA, THP II, and SA compared on the basis of nucleation times versus driving force. To nucleate at the same time, a higher driving force is required by THP:SA followed by SA. THP II nucleates with the greatest ease.

In Figure 5, the time for nucleation, τ_nuc_ (τ_50_ – τ_g_), is plotted against the driving force, showing that to reach the same nucleation time in the three systems, the lowest driving force is needed for theophylline and the highest for the cocrystal. In this sense, the nucleation of the cocrystal is more difficult than the nucleation of the pure compounds, with theophylline being the easiest to nucleate.

The induction time (τ) is the time lapse between the initiation of supersaturation and the very first observation of nucleation in the solution. This time includes a transient time of restructuring the clustering in solution, the nucleation time, and the time to grow to a detectable size. It is normally assumed that the induction time is governed by the nucleation time. However, in the present work, especially for the cocrystal, the growth time is not negligible. Accordingly, the actual nucleation time τ_nuc_ is calculated as τ_50 -_ τ_g_ and is related to the nucleation rate as given by eq 5
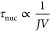
5where the volume is 20 mL. The nucleation rates calculated for each system are presented in Table 3. Overall nucleation rates are in the range of 5–175 m^–3^ s^–1^. These values are low but are of the same order of magnitude as values found for other organic substances when the evaluation is made by the classical nucleation theory.

**Table 3:** Nucleation Rates, *J*, for THP:SA, SA, and THP Estimated from the Nucleation Time According to eq 5

THP:SA			SA			THP		
*S*	τ_nuc_ (s)	*J* (m^–3^ s^–1^)	*S*	τ_nuc_ (s)	*J* (m^–3^ s^–1^)	*S*	τ_nuc_ (s)	*J* (m^–3^ s^–1^)
1.46	4121	12	1.30	9848	5	1.13	2905	17
1.57	1783	28	1.35	7041	7	1.18	2095	24
1.77	1289	39	1.38	2579	19	1.22	1707	29
2.16	1000	50	1.40	2563	20	1.27	1230	41
2.25	755	66	1.45	1810	28	1.31	1430	35
2.56	633	79				1.36	593	84
2.89	286	175						

The nucleation rate is commonly expressed in the form of the Arrhenius-type equation^32,33^
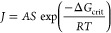
6where *A* (m^–3^ s^–1^) is a pre-exponential factor, accounting for the transport of molecules from the solution, and represents the abundance of molecules potentially available to add to the precritical cluster, raising the probability of nucleus formation.^34^*R* is the universal gas constant (J K^–1^ mol^–1^), *T* is the temperature, and Δ*G*_crit_ is the free-energy barrier that has to be surpassed in the formation of a cluster that is thermodynamically stable in the solution—a nucleus.

While the solute in the solid phase has a lower free energy than the solute in the supersaturated solution, the solute molecules at the surface of the solid phase have a higher free energy due to the unsatisfactory bonding at the surface. In the free-energy balance, the solid–liquid interfacial energy term (γ) is unfavorable and dominates at small sizes. For a spherical nucleus of critical size, the nucleation free energy (Δ*G*_crit_) per mole of nuclei (J mol^–1^), also called the nucleation work, can be given as eq 7
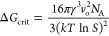
7where υ_o_ is the volume occupied by a molecule in the critical cluster.

**Figure 6:**
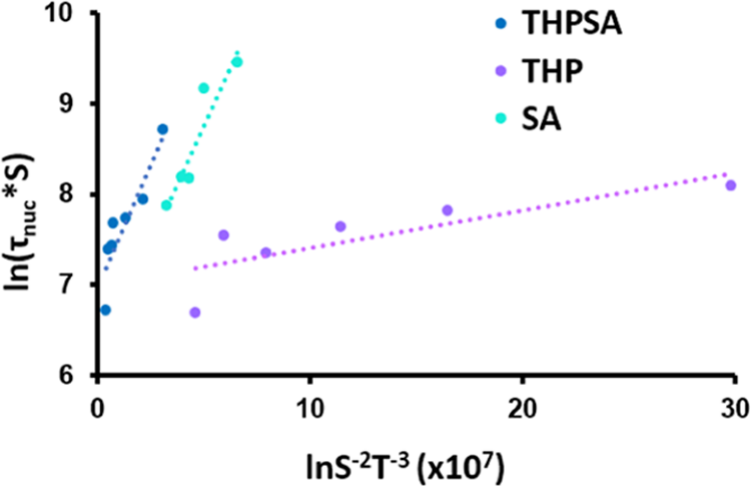
CNT plot for comparison of all three systems: THP:SA, SA, and THP II.

Combining eqs 5, 6, and 7, we obtain the relation that forms the basis for the classical nucleation plot, Figure 6, for determination of the interfacial energy and the pre-exponential factor
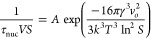
8

Looking at eq 8, the slope of ln(τ_nuc_ × *S*) versus ln *S*^–2^*T*^–3^ can be referred to as *B*
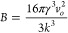
9

The slope of the cocrystal CNT graph is quite close to the slope of the salicylic acid CNT graph, while that of theophylline is clearly lower.

The THP:SA cocrystal consists of two chemical units, *Z* = 4 (number of formula units in a unit cell) and *Z*′ = 1, which is the number of asymmetric units, i.e., *Z* divided by the lowest multiplicity of Wyckoff positions. In the present work, the molecular volume of the cocrystal (υ_o_(cc)) is defined by eq 10, which corresponds to the value provided in the CSD according to the cocrystal cell parameters.^37^ The molecular volumes of pure SA and THP II are calculated by the same methodology.
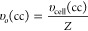
10

Accordingly, the cocrystal molecular volume is the volume occupied by a 1:1 assembly in the cocrystal of one molecule of each coformer and becomes 3.4558 × 10^–28^ m^3^.

**Figure 7:**
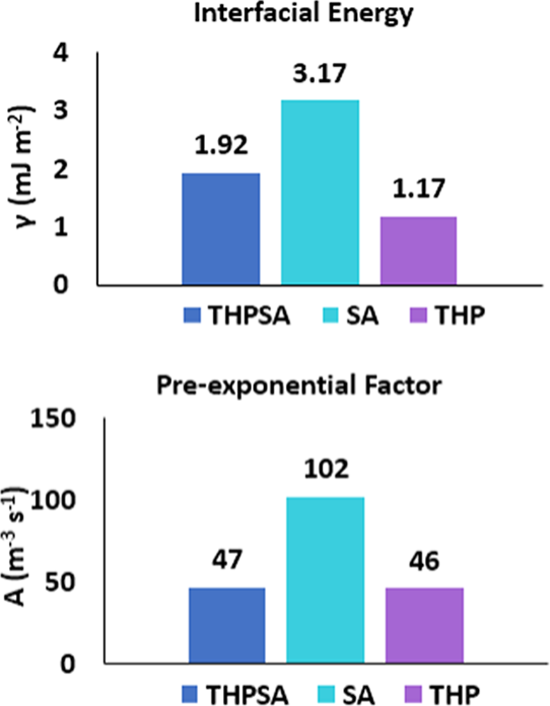
Top: Interfacial energies calculated from the slope of the ln (τ_nuc_ × *S*) versus ln*S*^–2^*T*^–3^ plot. Bottom: Calculated pre-exponential factors, *A*, from the intercept of CNT plots.

From the straight lines of Figure 6, the pre-exponential factor *A* and slope *B* are determined, and from the latter, the interfacial energy is determined. The resulting parameter values are presented in Figure 7. The THP:SA cocrystal has an intermediate interfacial energy value of 1.92 mJ m^–2^, whereas SA has the highest interfacial energy, γ, of 3.17 mJ m^–2^ and THP has the lowest value of 1.17 mJ m^–2^. THP:SA has a pre-exponential factor, *A*, value of 47 m^–3^ s^–1^, which is almost the same as the pre-exponential factor for THP II of 46 m^–3^ s^–1^ and much lower than that of SA, which has a value of 102 m^–3^ s^–1^. With regard to the statistical confidence of these values, note that a normal straight-line statistical analysis is not relevant since every data point along the straight CNT plot in Figure 6 is an average of a significant number of independent determinations. Using extensive survival theory analyses,^35,36^ it has been shown that for experimental conditions very much resembling those of the present work, the CNT parameters determined normally have sufficient confidence for the comparison of interfacial energies and pre-exponential factors.

Calculations of nucleation work, critical nucleus size (*r*_crit_), and number of molecules in the nucleus (*N*_crit_) are presented in the Supporting Information. The nucleation work (Δ*G*_crit_) according to eq 7 varies from below 1 kJ mol^–1^ to about 7 kJ mol^–1^ for the three systems. Notably, values as low as 1 kJ mol^–1^ are below the kinetic energy of the system, suggesting that there is no barrier to nucleation under those conditions. However, the experimental data show that the nucleation still requires a certain induction time and the nucleation rate is fairly low. This kind of inconsistency of the CNT has been reported in other studies. At a comparable driving force, e.g., 724 J mol^–1^, the maximum number of molecules to form a critical nucleus are required for the THP:SA cocrystal system—16 THP:SA dimer assemblies (16 THP and 16 SA molecules). SA requires 15 molecules, and THP requires just one molecule. Similar to the low nucleation work values, the very small number of molecules in the nucleus, also reported in other studies, is also referred to as inconsistency of the CNT. The volume of the critical nucleus (*v*_n_) at RT ln *S* = 724 J mol^–1^ is 5.68 × 10^–27^ m^3^ for the cocrystal, 2.36 × 10^–27^ m^3^ for SA, and 0.25 × 10^–27^ m^3^ for THP, correlating to the fact that the induction time is the longest for the cocrystal and the shortest for THP. It is more difficult to assemble a greater number of molecules into a nucleus. A plot of *v*_n_ versus RT ln *S* for all three systems is shown in the Supporting Information.

## Discussion

The slope of the CNT plots in Figure 6 decreases in the order of THP:SA > SA > THP but is very similar for SA and the cocrystal. However, SA has a much higher interfacial energy than the cocrystal, 3.17 versus 1.92 mJ m^–2^. The reason is the difference in molecular volume. In eq 9, the slope *B* ∝ γ^3^υ_o_^2^. The THP:SA cocrystal has a molecular volume over twice that of SA. Accordingly, the very similar slope leads to a higher interfacial energy for SA because of a smaller molecular volume. It is reasonable from a physical chemistry point of view that the cocrystal has an interfacial energy between the values for the pure compounds, since the surfaces of the cocrystal should expose a combination of unsatisfactory bonds to SA and THP molecules. Notably, the value obtained for the cocrystal interfacial energy, 1.92 mJ m^–2^, is almost the same as the geometric mean of the two pure compound interfacial energies (eq 11), yielding a value of 1.93 mJ m^–2^.
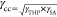
11

In the CNT plot, the line for SA has a higher slope than that for THP, which is reflected in the higher interfacial energy of SA. The larger molecular volume of THP, Table 4, is not sufficient to alter this relation.

**Table 4:** Molecular Volume (υ_o_) of the Three Systems According to the CSD and γ^3^υ_o_^2^ of eq 9, Where γ is the Interfacial Energy

	molecular volume		
	Å^3^	m^3^ (×10^–28^)	γ^3^υ_o_^2^ (×10^–55^)
THP:SA	345.58	3.4558	8.45
SA	158.83	1.5883	8.04
THP II	200.35	2.0035	0.63

The derivation of eq 8 starts from establishing the free energy of formation of a cluster (used here to denote an entity having a crystalline structure) from molecules dissolved in the supersaturated solution as is given in standard textbooks on the subject. The free energy of the cluster is the volume times the free energy per unit volume of the crystalline structure minus the free energy of the cluster surface. The surface term corrects for the fact that molecules at the surface of the cluster have a higher free energy, because of absence of bonding, compared to the molecules inside the crystalline structure. The free-energy difference per molecule between a molecule in the lattice and a molecule in the supersaturated solution is approximated as *kT* ln *S*. In the conversion of the crystal structure free-energy gain upon formation of the cluster per unit volume (Δ*G*_υ_) into the corresponding value per molecule, the molecular volume (υ_o_) is introduced as follows
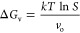
12

where *k* is the Boltzmann constant in J K^–1^, *T* is the temperature in K, and *S* is the supersaturation ratio. According to the equation, for the same supersaturation driving force, the rate of nucleation decreases with increasing molecular volume because the free-energy gain per unit volume of the cluster at equal driving force (J/molecule) decreases with increasing molecular volume. It can be discussed what is an appropriate molecular volume for the cocrystal, and this will influence the value determined for the interfacial energy but not the slope value of *B* (eq 9) and not the driving force required to reach a specific induction time.

Alternative methods to extract nucleation parameters from the experimental data are explored in the Supporting Information. The results show that the determination of the interfacial energy is quite insensitive to the method used, while the value of the pre-exponential factor does change. The pre-exponential factor, *A*, is related to physical properties according to eq 13 for volume-diffusion-controlled nucleation and according to eq 14 for interface-transfer-controlled nucleation^33^
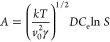
13
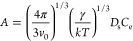
14where *v*_o_ is the molecular volume (m^3^), *D* is the diffusivity *(*and *D*_s_ is the surface diffusivity)^38,39^ (m^2^ s^–1^), γ is the interfacial energy (J m^–2^), *C*_e_ is the solubility (i.e., in the present work at 10 °C) in molar concentration (mol m^–3^), and *A* is obtained in m^–3^ s^–1^. In volume-diffusion-controlled nucleation, the controlling step is transport of growth units over the hydrodynamic boundary layer around the particle in the liquid. The attachment frequency is the product of monomer diffusion flux and the surface area of the nucleus. For interface-transfer-controlled nucleation, the rate-controlling step is the transport of growth units over the surface in an adsorbed state to the location of lattice integration. Unfortunately, little is known about the surface diffusion transport in this type of system, and as a first rough approximation, the corresponding diffusivity is assumed to be the same as in volume diffusion.^39^

In the present study, the diffusivity of the pure compounds has been estimated by the Wilke and Chang equation^38,40^
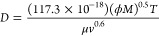
15where *D* is the diffusivity in m^2^ s^–1^, *M* is the molecular weight of the solvent in kg kmol^–1^, *T* is the temperature in K, μ is the solvent viscosity in kg m^–1^ s^–1^, *v* is the solute molar volume in m^3^ kmol^–1^, and ϕ is the association factor for the solvent, which is 1 for chloroform. *D* values are presented in Table 5.

**Table 5:** Comparison of the Relationship between Pre-Exponential Factors and Diffusivity for Three Solutes in Chloroform at 10 °C According to eqs 13 and 14^a^

	molar volumes (m^3^ kmol^–1^)	*D* (m^2^ s^–1^) (×10^–10^)	*C*_e_ (mol m^–3^)	γ (mJ m^–2^)	*A*, eq 13 (m^–3^ s^–1^) (×10^8^)	*A*, eq 14 (m^–3^ s^–1^) (×10^9^)	*A*, exp. (m^–3^ s^–1^)
THP:SA	0.1200^b^	1.06^b^	11.70	1.92	9.30	2.38	47
SA	0.0958	1.21	67.93	3.17	104.64	22.0	102
THP II	0.1200	1.06	17.36	1.17	30.49	2.76	46

a An *S* value of 1.20 was chosen for the sake of comparison. The pre-exponential factor, *A*, calculated from induction time experiments is included. Diffusivity of the cocrystal used in eqs 13 and 14 is that of THP.

b Cocrystal diffusivity is that of the slowest diffusing component, i.e., THP, and therefore the molar volume used in eq 15 is also that of THP.

For the cocrystal, it is assumed that the transport rate is governed by the component having the lowest diffusivity, i.e., THP; thus, in the formation of the cocrystal nucleus, the diffusivity (*D*) in eq 13 is taken as that of THP. In eq 13, the cocrystal nucleus radius is governed by thermodynamics and, accordingly, the molecular volume term (υ_o_) remains the same as that of the cocrystal. Likewise, for eq 14, the diffusivity in the expression for the cocrystal system is taken as that of THP—the rate-limiting molecule. Since the molecular volume, *υ*_o_, in eq 14 relates to the diffusion flux from the f* attachment frequency parameter, involving a jump comparable to the diameter of the attaching molecule, for interface-transfer-controlled nucleation is taken as that of THP. The driving force for the transport of THP in the formation of the cocrystal nucleus is accordingly the difference in the THP concentration of the actual solution and that of a solution in equilibrium with the cocrystal solid phase, and *C*_e_ (mol m^–3^) is taken as the latter value. For THP and SA nucleation, *C*_e_ is the equilibrium concentration of the pure components (mol m^–3^). *S* = 1.20 is used for the sake of comparison in calculations of eqs 13 and 14.

The pre-exponential factors calculated by eqs 13 and 14 are presented in Table 5. Obviously, these values are much higher than those found experimentally, which agrees with essentially all previous studies and has been recognized as an important question mark when it comes to the quantitative validity of the classical nucleation theory.^26,33,41^ However, overall, the values follow the same trend as those calculated from the experimental induction time data. The value for SA is clearly higher than the values for the other two systems, even though the values calculated by eqs 13 and 14 are more pronounced in this respect compared to the experimental data. For the cocrystal and for theophylline, the experimental values are very similar, and this is very well captured by eq 14 but somewhat less so by eq 13. In eq 13, *A* is directly proportional to diffusivity and solubility, both of which are the highest for the SA system. *A* is inversely proportional to the interfacial energy, which is the highest for SA. These parameters counteract the fact that the molecular volume is the lowest for SA. In eq 14, the dependence on these parameters is overall similar.

Looking at nucleation difficulty, THP is the easiest to nucleate because of its lowest interfacial energy. Since THP:SA has a medium interfacial energy, it would be expected to be the second easiest to nucleate; however, this is not the case. SA is the second easiest to nucleate due to its high kinetic factor, which overcomes the effect of the large interfacial energy. THP:SA is the most difficult to nucleate due to an intermediate interfacial energy value and a low pre-exponential factor, similar in value to that of THP.

It has been proposed that interfacial energy would decrease with increasing solubility,^43^ even though the evidence of validity for organic systems in different solvents is less clear than that for inorganic compounds in aqueous solution. The experimentally determined interfacial energy (mJ m^–2^) from induction time experiments decreases from SA to THP:SA to THP II; however, the molar solubility decreases in the order of SA to THP II to THP:SA.

## Conclusions

Overall, the experimental results suggest that the driving force required for equal induction times of the three solid phases increases in the order THP II, SA, and THP:SA. Interfacial energies calculated for the systems increase in the order THP II, THP:SA, and SA. The data shows that there is nothing inherently different between the nucleation of pure components and the nucleation of the cocrystal. Pre-exponential factors (*A*) calculated from induction time experiments exhibited a pattern of increasing values in the order of THP, THP:SA, and SA, with very similar values for THP:SA and THP. The experimental values of *A* agree reasonably with *A* values calculated from theoretical expressions for volume-diffusion- and surface-integration-controlled nucleation. Despite moderate interfacial energy, the nucleation work of THP:SA is the highest because of the highest molecular volume. Pre-exponential factors seem to be dominated by the effect of molecular volume and solubility. The onset of nucleation is clearly different between the three systems. There is a significant difference in how the nucleation evolves into a full cloud point for the three systems. For the cocrystal, it takes 1–3 h from the very first observation of a nucleation event to full opacity, depending on supersaturation. For THP II, full opacity is reached within a period of 3–6 min, and for SA, it takes 10–30 s. The speed increases in the same order as the solubility and the growth rate parameter. Since for the cocrystal there must be an arrangement of two different molecules, there is an added kinetic barrier, and higher driving forces are required for nucleation rates similar to the pure components.

## Supporting Information Available

Details of solubility determination and solid-phase characterization techniques; PXRD patterns; DSC curves; SEM micrographs; analysis of nucleation work (Δ*G*_crit_) and critical nucleus size versus driving force; table of τ_g_ values; calculations of the pre-exponential factor; and comparison of various methods to calculate *J* (PDF)
