# Supplementary material for: Nucleation of the Theophylline:Salicylic Acid 1:1 Cocrystal
Source: Cryst Growth Des. 2021 Apr 1;21(5):2711–9. doi: 10.1021/acs.cgd.0c01594 (PMC8816349; doi:10.1021/acs.cgd.0c01594)
Supplement: Supplementary file 1 — cg0c01594_si_001.pdf [file cg0c01594_si_001.pdf]

Nucleation of the Theophylline:Salicylic Acid 1:1 Cocrystal – ESI

Hannah McTague[1] and Åke C. Rasmuson*[1,2]

[1]Synthesis and Solid State Pharmaceutical Centre (SSPC), Bernal Institute, Department of Chemical and Environmental Science, University of Limerick. Limerick V94 T9PX, Ireland.

[2]Department of Chemical Engineering and Technology, KTH Royal Institute of Technology, SE-100 44 Stockholm, Sweden.

Solubility determination

The solubility was determined for SA, THP II and THP:SA in chloroform at the nucleation temperature 10 °C by gravimetric method. The structure of the solid forms for each used were obtained prior to solubility experiments by PXRD. THP II and SA were used as received from Sigma-Aldrich where THP II was identified as the solid form. THP:SA was used directly after synthesis in a chloroform slurry and identified by PXRD. Solutions containing each component were allowed to equilibrate with excess solid in a sealed 20 mL vial for 72 h. The 20 mL vials were stirred with small PTFE magnetic stir bars at 400 rpm. A reference vial containing just chloroform solvent was placed in the bath besides the samples and checked by digital thermometer to ensure the temperature in the vials remained at the nucleation temperature. Samples were stopped stirring and allowed to settle for 1 hour. 5 mL or 10 mL samples were then taken of the liquid and filtered by 0.2 µm PTFE filters into pre weighed vials with lids. The lids were then removed and total evaporation of the chloroform ensued. After 72 hours the vials containing dried solid are weighed once and then again 2 hours later. If there is no change in weight the chloroform has completely evaporated. Repeat experiments were taken for each the SA, THP II and cocrystal under each set of conditions. The PXRD pattern of the excess solid from the settled solution was recorded to ensure that the solubility of the desired polymorph was determined.

Solid phase characterisation

PXRD data were collected in reflection mode with an Empyrean diffractometer (PANalytical, Phillips) equipped with CuKα1,2 radiation (γ = 1.5406 Å) operating at 40 kV and 40 mA at room temperature. Samples were scanned between 2θ values of 5 and 40° at a step size of 0.01313° 2θ/s, 73 s per step.

Differential scanning calorimetry (DSC) was performed on a Netsch Polyma 214 DSC. Samples were analysed in a nitrogenous environment with a temperature ramp rate of 10 °C min^{-1} over a temperature scan range from 20 and 310 °C. Crystals were isolated from solvent with Whatman filter paper. 5-7 mg of crystals were added to concavus aluminium pans which were sealed using a crimping press and then the lid was pierced. The instrument was calibrated using samples of indium and lead.

Thermogravimetric analysis (TGA) was carried out under nitrogen using TGA instrument TA Q50 V20.13 Build 39. Samples were placed on platinum pans and heated up to 500 °C at a ramp rate of 20 °C min^{-1}.

For single crystal x-ray diffraction (SC-XRD) analysis cocrystal material was dissolved in chloroform to create a saturated solution. A crystal of suitable quality was obtained by slow evaporation crystallisation and analysed by SC-XRD. The unit cell parameters were consistent with that on record in the CSD.[1]

For Scanning Electron microscopy (SEM), following visible nucleation of vials of all three systems; TPH:SA, THP II and SA over a variety of conditions the slurry was isolated immediately. Analysis of the solid isolated from the slurry was carried out using SEM on the Jeol CARRYSCOPE, coated in gold by a 30 second sputter to ensure a fine coating and minimise ionization. Samples isolated from the same slurries were analysed also by PXRD to identify solid forms.

The THP:SA cocrystal was successfully synthesized as shown by subsequent physical characterisation including PXRD and DSC. The THP:SA cocrystal diffractogram was identical to a previously reported powder pattern for the cocrystal entered to the Cambridge structural database (CSD) under the reference code KIGLES01 (**Figure 1**). The PXRD pattern of the cocrystal is distinctly different to diffractograms of pure SA and the two low-temperature forms of pure THP.[1] (**Figure 2**). The diffractogram patterns of the samples taken from the bulk powder following synthesis revealed peaks only corresponding to THP:SA cocrystal, proving that conversion is completed within 72 h.

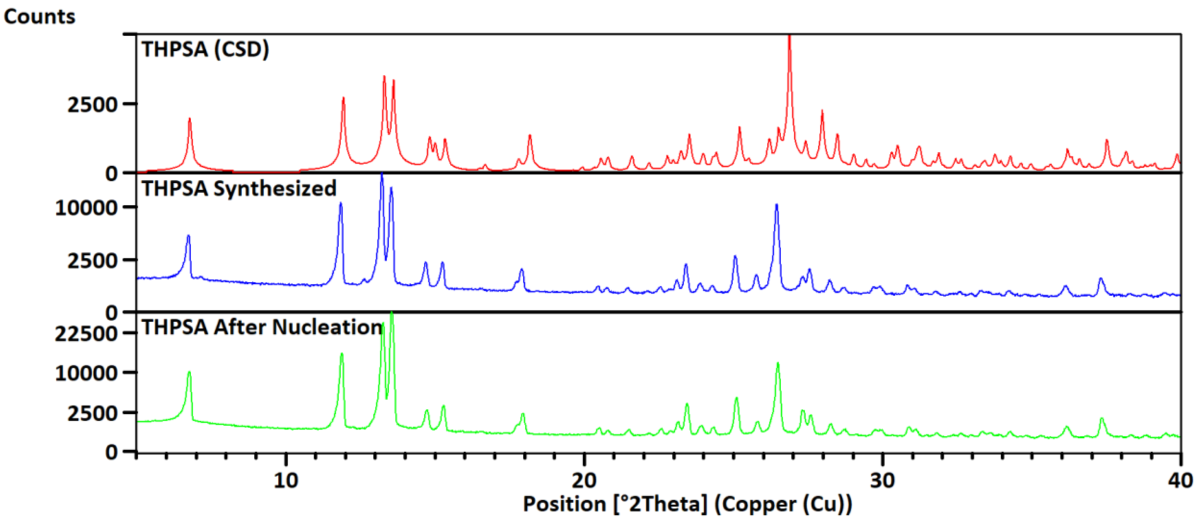

Figure 1 Diffractogram of previously reported THP:SA cocrystal pattern in CSD (Ref. Code: KIGLES01) (red), synthesized THP:SA cocrystal (blue) and THP:SA isolated from vial following induction time experiments in chloroform at 10 °C.

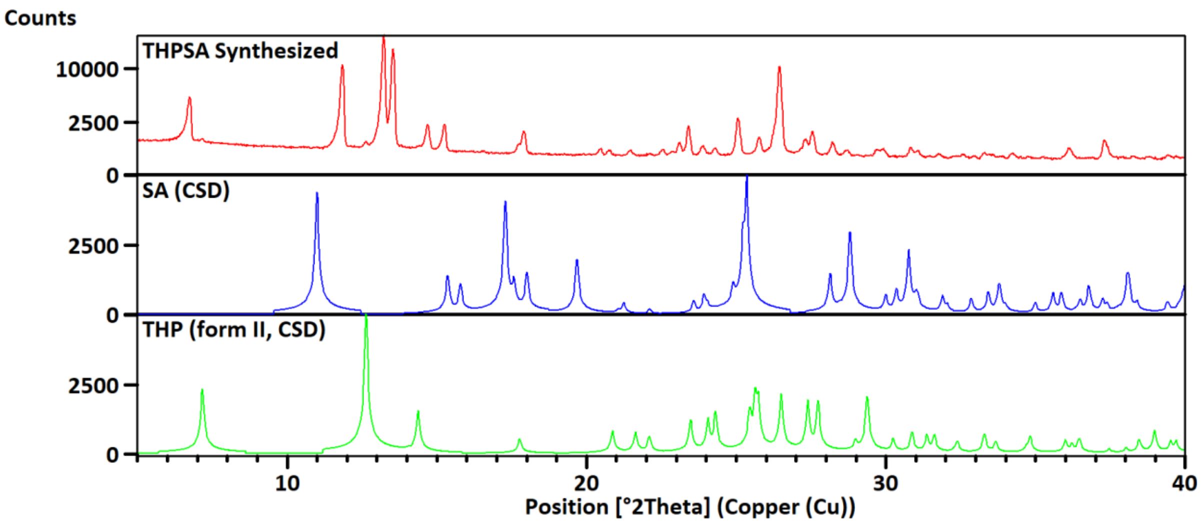

Figure 2 THPSA cocrystal diffractogram versus the two pure coformers THP II (CSD Ref. code BAPLOT01) and SA (CSD Ref. code SALIAC).

The THP II powder was analysed as received and identified as polymorphic form II (CSD Ref. code BAPLOT01). THP II solid isolated following induction time experiments was also identified as form II as per the diffractogram in **Figure 3**.

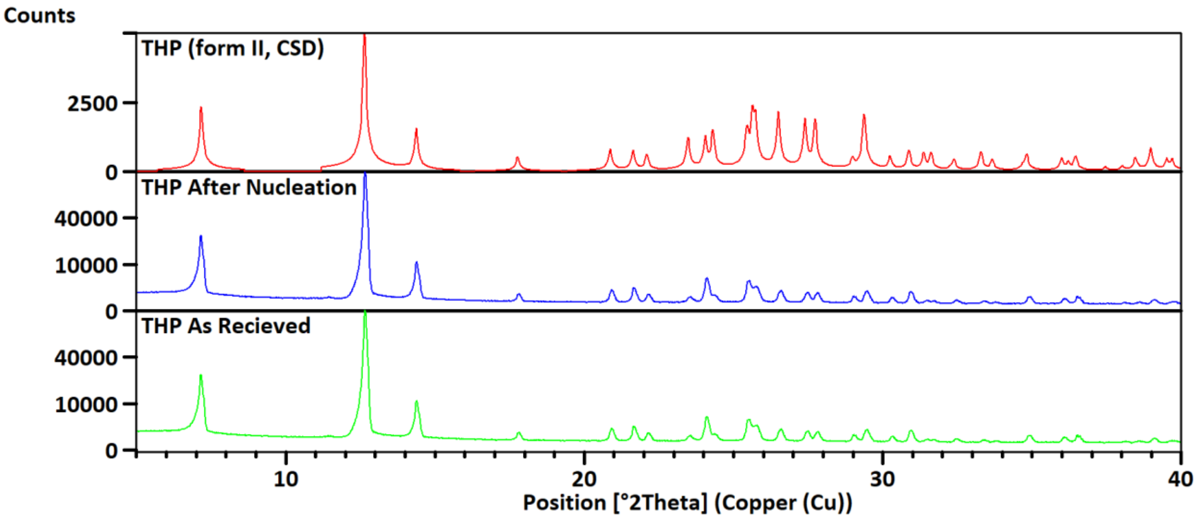

Figure 3 Diffractogram of THP form II powder pattern from CSD (Ref. code BAPLOT01) compared with THP II as received and THP II isolated following induction times in chloroform at 10°C.

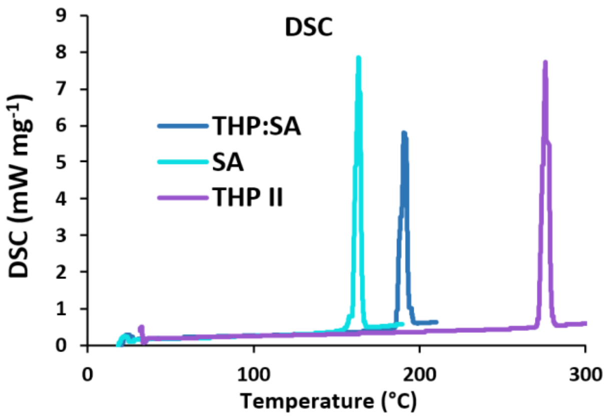

Figure 4 DSC curves obtained for THP:SA, THP II and SA using a ramp rate of 10°C min^{-1}. The temperature corresponding to the onset of each endotherm peak is labelled.

The DSC curve in **Figure 4** shows the melting point of the cocrystal is between that of the API and coformer. The melting point of THP:SA is 186.2 °C, SA is 158.8 °C and THP II is 272.0 °C. A DSC curve previously reported in the literature of THP:SA shows the onset of melting as 188.5 °C[1]. One main difference between the experiments is the different heating rates used as the work reported previously operated at 5 °C min^{-1} whereas the data in the current work was collected at 10 °C min^{-1}, sample mass also effects detection of melting peaks along with different instruments. The same cocrystal sample for which we ran DSC was also analysed by PXRD and matched the PXRD pattern reported by the authors of the previous work outlined above[1]. The THP form II melting point is 273 °C which matches the data from the same paper. The same previous work also reports a melting point onset for SA of 159 °C, PXRD patterns supported this identification of the SA samples.

Table 1 DSC data calculated by Netsch Proteus software

	**No. Samples**	**Onset of Endotherm (°C) (Average)**	**Enthalpy of Fusion (J g^{-1})**	**Molar Heat of Fusion (KJ mol^{-1})**	**Melting point from Zhang *et. al*[1] (°C)**
**THP:SA**	4	186.2	145.1	46.142	188.5
**SA**	1	158.8	178.5	24.633	159.0
**THP II**	3	272.0	167.6	30.168	273.0

THP:SA presents the lowest enthalpy of fusion of 145.1 J g^{-1}. This value is lower than the enthalpy of fusion of both THP II and SA. SA has the highest enthalpy of fusion at 178.5 J g^{-1}.

The cocrystal is in the form of needles (**Fig. 5(a**)), SA is also in the form of needles (**Fig. 5 (b)**) and THP II is plate-like (**Fig. 5(c)** and (**d**)).

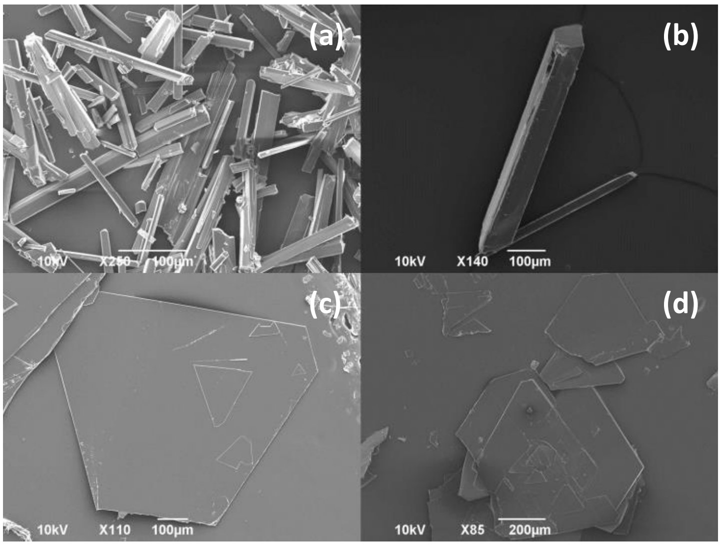

Fig. 5 (a) THP:SA crystals obtained by evaporation from chloroform. **(b)** Crystals of SA obtained by evaporation from chloroform **(c)** and **(d)** THP II crystals obtained by evaporation from chloroform.

For a spherical nucleus of critical size, the nucleation free energy (*ΔG_{crit}*) per mole of nuclei (J mol^{-1}), also called the nucleation work, in **ESI Eq [1]** is given as

$$\Delta G_{crit} = \frac{16\pi\gamma^3 v_o^2 N_A}{3(kTlnS)^2} \qquad \textbf{[1]}$$

In **Figure 5** it is shown that the nucleation work, ΔG_{crit}, is the highest for THP:SA closely followed by that of SA. The nucleation work is much lower for THP II. However, notably at higher supersaturation the nucleation work is even less than the kinetic energy of the system: 3/2RT and should proceed spontaneously. However, it doesn't showing one of the known weaknesses of the CNT.

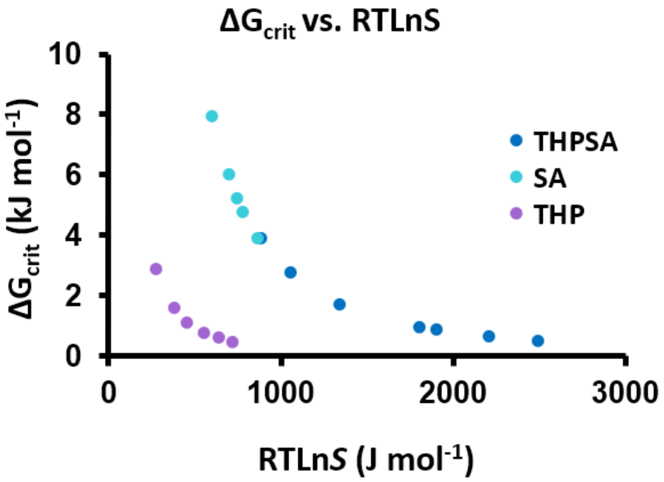

Figure 5 ΔG_{crit} versus RTLn*S* for all three solid phases.

The critical radius size for THP:SA, THP and SA under different driving forces has been calculated according to **ESI Eq [2]** and presented in **Figure 6**.

$$r_{crit} = \frac{2v_o\gamma N_A}{RTlnS} \qquad \textbf{[2]}$$

From **ESI Eq [2]** the number of molecules per critical nucleus can be estimated[2]:

$$N_{crit} = \frac{4\pi r_{crit}^3}{3v_o} \qquad \textbf{[3]}$$

Where r_{crit} is critical radius (m), v_o is molecular volume (m^3) as shown in **Table 4 (main text)**, γ is interfacial energy (J m^{-2}) and N_A is Avogadro's number and N_{crit} is the number of molecules per critical nucleus. At comparable driving force the THP:SA critical nucleus has a larger radius than SA and THP has the smallest r_{crit}. THP:SA requires a slightly lower number of molecules than SA to make up the nucleus. As seen in **Figure 6** at comparable driving force, 724 J mol^{-1} (*S*=1.36), the least number of molecules to form a nucleus is required for the THP system (1 molecule), SA requires 15 molecules and the most required by THP:SA (16 THP:SA heterodimers which includes 16 THP and 16 SA molecules).

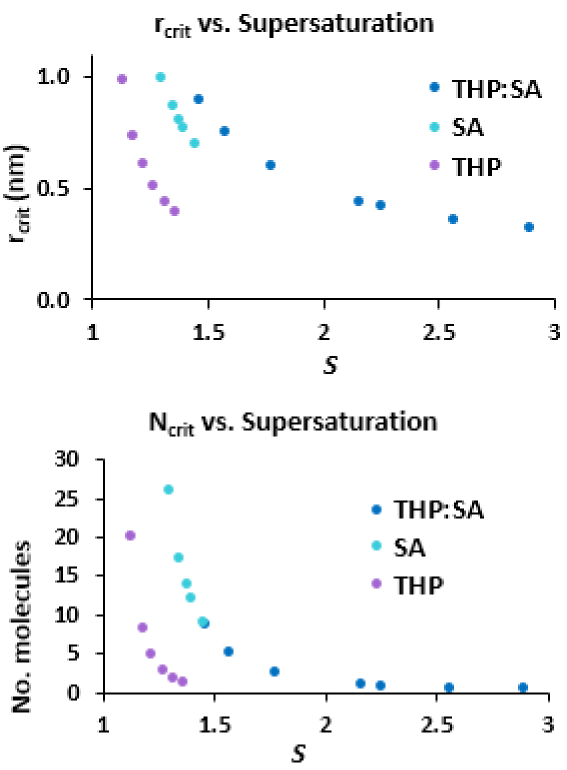

Figure 6(a) Critical nucleus size (r_{crit}) in nm versus supersaturation for THP:SA cocrystal, SA and THP II systems. The largest critical nucleus size for nucleation is exhibited by THP:SA followed by SA whereas THP displays the smallest r_{crit}. (b) $N_{crit,}$ the number of molecules per critical nucleus versus supersaturation. For the cocrystal molecules means heterodimers

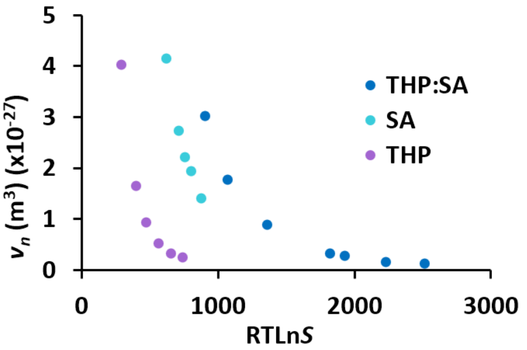

Figure 7 Volume of the critical nucleus (v_n) versus driving force RTLnS (J mol^{-1}) for the three systems.

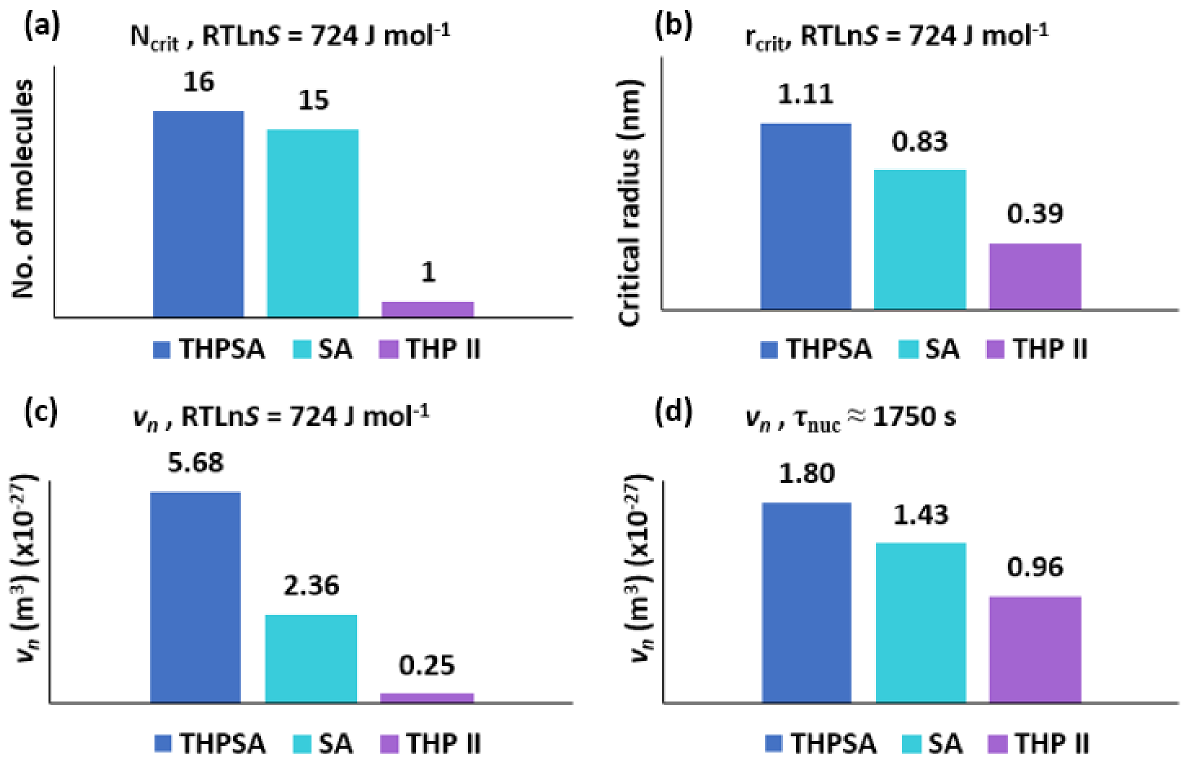

Figure 8 (a) (b) and **(c)** Shown respectively are $N_{crit,}$, $r_{crit,}$ and v_n for THP:SA, SA and THP at equal driving force of 724 J mol^{-1}. **(d)** The volume of the critical nucleus, v_n is compared for the three systems at the same nucleation time of ~1750 seconds.

Table 2 τ_g from the Poisson distribution fit and the τ_g defined as the first experimentally observed induction time point on that distribution.

THP:SA			SA			THP		
S	**τ_g (Poisson)**	**τ_g (first point)**	***S***	**τ_g (Poisson)**	**τ_g (first point)**	***S***	**τ_g (Poisson)**	**τ_g (first point)**
1.46	2959	3205	1.30	681	338	1.13	1732	1980
1.57	1889	1497	1.35	59	205	1.18	1733	1471
1.77	1611	1216	1.38	-934	195	1.22	920	710
2.16	827	735	1.40	29	188	1.27	1126	870
2.25	1199	992	1.45	298	210	1.31	531	530
2.56	777	612	-	-	-	1.36	357	445
2.89	534	530	-	-	-	-	-	-

Table 3 presents estimation of preexponential factors using an approach where the diffusivity of the cocrystal is taken as the geometric mean of THP and SA diffusivities[3]:

$$D_{AB} = \sqrt{D_A . D_B} \qquad \textbf{[4]}$$

In this case it would be assumed that there are cocrystal assemblies diffusing throughout the liquid and therefore the C_e term used in this calculation would be the concentration of the cocrystal in equilibrium with the solid cocrystal phase and the value of v_o used would be the molecular volume of the cocrystal.

Table 3 *A* calculated from **Eq [13]** and **Eq [14]**[4] from the main text for THP:SA using the cocrystal diffusivity as a geometric average (**ESI Eq [4]**) and the molecular volume of the cocrystal. The bottom row represents the new values calculated when the diffusivity of the rate limiting step – THP is used in both cases and the molecular volume of THP is used in the surface integration version.

**THP:SA cocrystal**	A, **[13]** (volume diffusion) ($x10^{10}$) (m^{-3} s^{-1})	A, **[14]** (interface transfer) ($x10^{09}$) (m^{-3} s^{-1})
***D* from ESI Eq [4]and v_o of cocrystal**	1.67	2.13
**Manuscript estimations assuming THP to be the limiting component (*D* of THP for [13] *D* and v_o of THP in [14])**	1.56	2.38

Methods for calculating *J*

Below are 4 variations of methods used to calculate the nucleation parameters *A*, and γ form the nucleation rate parameter *J*. Method 1 is the same as what is presented in the main body of work and the others are for comparison.

Method 1:

$$J = \frac{1}{\tau_{nuc}\, V}$$

Where $\tau_{nuc} = \tau_{50} - \tau_g$ and τ_g is the first point.

Method 2[5]:

$$P(\tau) = 1 - e^{\left(-JV(\tau-\tau_g)\right)}$$

Where τ, τ_g and *J* are estimated from the best fit of the Poisson distribution to the experimental distribution.

Method 3:

$$P(\tau) = 1 - e^{\left(-JV(\tau-\tau_g(first\ point))\right)}$$

Where τ, and *J* are estimated from the best fit of the Poisson distribution to the experimental distribution but τ_g is defined as the first point.

Method 4:

$$J = \frac{1}{\tau_{50}\, V}$$

Where no growth time is accounted for and *J* is estimated from the experimental induction times τ_{50}.

The values of the parameters used for each method are presented in **Table 5 – 8**.

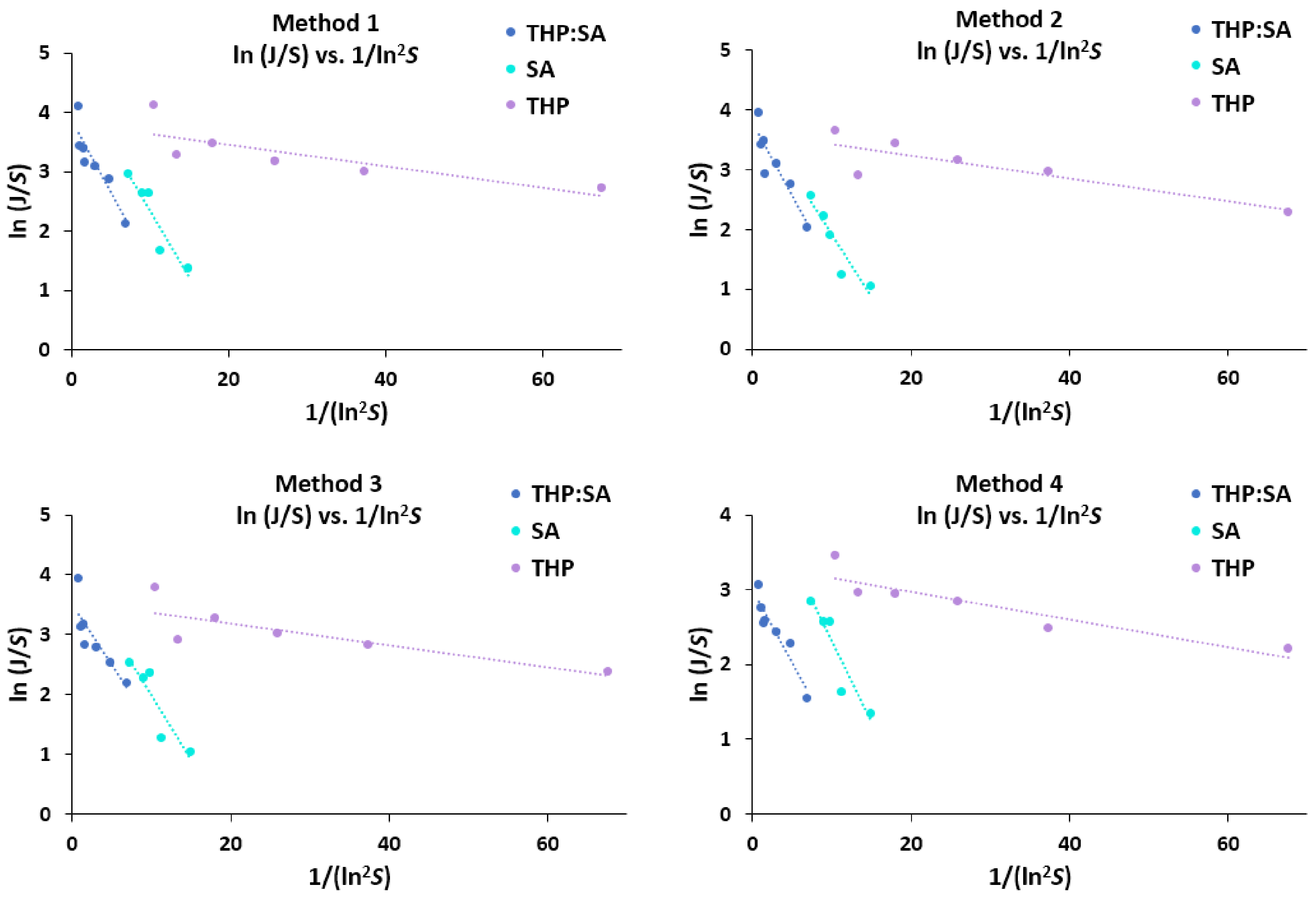

Figure 9 CNT plots for Methods 1-4.

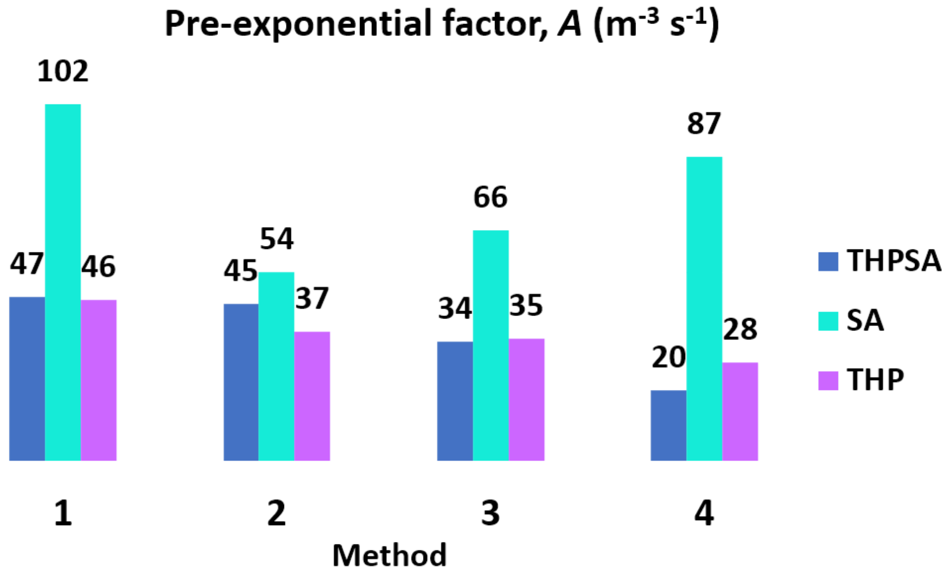

Figure 10 Pre-exponential factor calculated for the 3 systems by the 4 different methods outlined above.

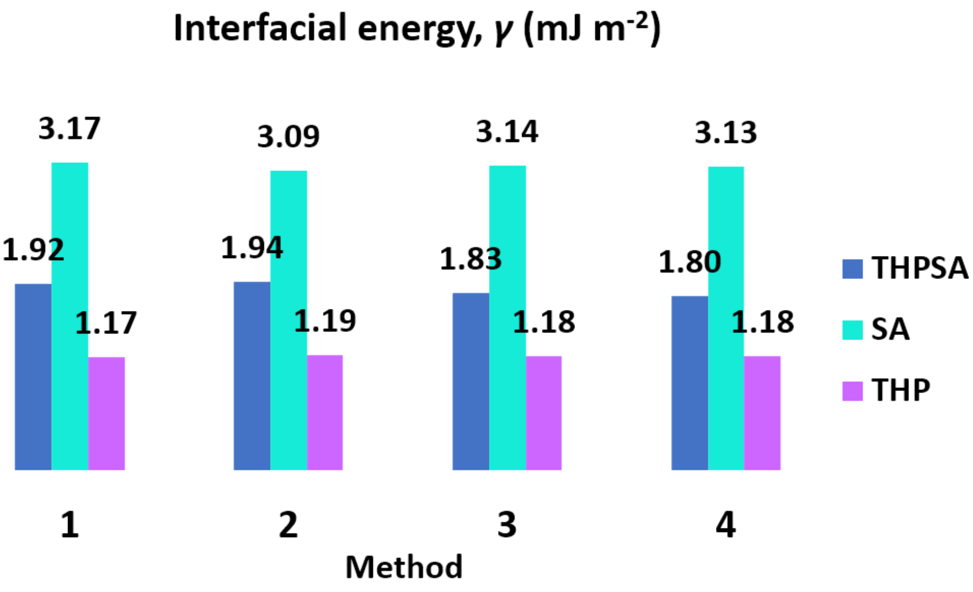

Figure 11 Interfacial energy calculated for the 3 systems by the 4 different methods demonstrated little change between methods.

The comparison reveals that in the SA system the Poisson distribution overestimates τ_{50} which, when accompanied by small τ_g values, results in lower than expected *J* values with respect to *J* estimated from the experimental τ_{50} values when a growth time is not considered. This led to a lower pre-exponential factor for SA by the method using Poisson distributions than expected. The same effect was not seen for THP:SA or THP as the Poisson distribution is a better fit to those experimental data. As the Poisson distribution does not represent the experimental data well in the SA system, the nucleation rates (*J*) have been estimated from the inverse of τ_{nuc} and *V* (eq. 6). This method was also utilised in the other systems for consistency. At the same driving force THP:SA has the lowest nucleation rate followed by SA and then THP which has the highest nucleation rate.

Table 4

S	Method 1			
**THPSA**	**τ_{nuc} (s)**	**τ_{50} (s)**	**τ_g**	**J (m^{-3} s^{-1})**
**1.46**	4121	7326	3205	12.13
**1.57**	1783	3280	1497	28.04
**1.77**	1289	2505	1216	38.79
**2.16**	1000	1735	735	50.00
**2.25**	755	1747	992	66.23
**2.56**	633	1245	612	78.99
**2.89**	286	816	530	174.83
**THP**				
**1.13**	2905	4885	1980	17.21
**1.18**	2095	3566	1471	23.87
**1.22**	1707	2417	710	29.29
**1.27**	1230	2100	870	40.65
**1.31**	1430	1960	530	34.97
**1.36**	593	1160	445	84.32
**SA**				
**1.30**	9848	10186	338	5.08
**1.35**	7041	7246	205	7.10
**1.38**	2579	2774	195	19.39
**1.40**	2563	2751	188	19.51
**1.45**	1810	2020	210	27.62

Table 5

S	Method 2			
**THPSA**	**τ_{nuc} (s)**	**τ_{50} (s)**	**τ_g**	**J (m^{-3} s^{-1})**
**1.46**	3112	6071	2960	11.14
**1.57**	1408	3297	1889	24.62
**1.77**	879	2490	1611	39.43
**2.16**	869	1696	827	39.88
**2.25**	469	1669	1200	73.88
**2.56**	441	1219	778	78.6
**2.89**	231	765	534	150.02

**THP**				
**1.13**	3123	4855	1732.43	11.1
**1.18**	1515	3247	1732.57	22.88
**1.22**	1206	2126	920.13	28.73
**1.27**	889	1932	1042.96	39.64
**1.31**	1445	1976	531.32	23.98
**1.36**	660	1017	357.12	52.5
**SA**				
**1.30**	9312	9993	681.44	3.72
**1.35**	7471	7530	59.37	4.64
**1.38**	3733	2798	-934.40	9.28
**1.40**	2716	2745	29.21	12.76
**1.45**	1833	2043	210.00	18.91

Table 6

**S**	**Method 3**			
**THPSA**	**τ_{nuc} (s)**	**τ_{50} (s)**	**τ_g**	**J (m^{-3} s^{-1})**
**1.46**	2653	5858	3205	13.06
**1.57**	1759	3256	1497	19.70
**1.77**	1223	2439	1216	28.35
**2.16**	956	1691	735	36.24
**2.25**	652	1644	992	53.16
**2.56**	593	1205	612	58.42
**2.89**	235	765	530	147.69
**THP**				
**1.13**	2862	4842	1980	12.11
**1.18**	1739	3210	1471	19.92
**1.22**	1385	2095	710	25.02
**1.27**	1038	1908	870	33.39
**1.31**	1446	1976	530	23.96
**1.36**	576	1021	445	60.16
**SA**				
**1.30**	9627	9965	338	3.60
**1.35**	7316	7521	205	4.74
**1.38**	2401	2596	195	14.43
**1.40**	2555	2743	188	13.56
**1.45**	1924	2134	210	18.01

Table 7

**S**	**Method 4**	
**THPSA**	**τ_{50} (s)**	**J (m^{-3} s^{-1})**
**1.46**	7326	6.83
**1.57**	3280	15.24
**1.77**	2505	19.96
**2.16**	1735	28.82
**2.25**	1747	28.62
**2.56**	1245	40.16
**2.89**	816	61.27

**THP**		
**1.13**	4885	10.24
**1.18**	3566	14.02
**1.22**	2417	20.69
**1.27**	2100	23.81
**1.31**	1960	25.51
**1.36**	1160	43.10
**SA**		
**1.30**	10186	4.91
**1.35**	7246	6.90
**1.38**	2774	18.02
**1.40**	2751	18.18
**1.45**	2020	24.75

References

(1) Zhang, S.; Chen, H.; Rasmuson, Å. C. Thermodynamics and Crystallization of a Theophylline-Salicylic Acid Cocrystal. *CrystEngComm* **2015**, *17* (22), 4125–4135. https://doi.org/10.1039/c5ce00240k.

(2) Yang, H.; Svä, M.; Zeglinski, J.; Rasmuson, Å. C. Influence of Solvent and Solid-State Structure on Nucleation of Parabens. **2014**. https://doi.org/10.1021/cg500449d.

(3) Rehfeldt, S.; Stichlmair, J. Measurement and Calculation of Multicomponent Diffusion Coefficients in Liquids. *Fluid Phase Equilib.* **2007**, *256* (1–2), 99–104. https://doi.org/10.1016/J.FLUID.2006.10.008.

(4) Kashchiev, D.; van Rosmalen, G. M. Review: Nucleation in Solutions Revisited. *Cryst. Res. Technol.* **2003**, *38* (78), 555–574. https://doi.org/10.1002/crat.200310070.

(5) Jiang, S.; Ter Horst, J. H. Crystal Nucleation Rates from Probability Distributions of Induction Times. *Cryst. Growth Des.* **2011**, *11* (1), 256–261. https://doi.org/10.1021/cg101213q.
